# Review of HIV response in Pakistan using a system thinking framework

**DOI:** 10.3402/gha.v8.25820

**Published:** 2015-03-30

**Authors:** Muhammad Ahmed Abdullah, Babar Tasneem Shaikh

**Affiliations:** 1 Health Services Academy, Islamabad, Pakistan; 2 Community and Family Medicine, Shifa Tameer-e-Millat University, Islamabad, Pakistan; 3 Health Systems & Policy, Health Services Academy, Islamabad, Pakistan

**Keywords:** HIV/AIDS, Pakistan, health system, building blocks

## Abstract

Pakistan has moved from a ‘low prevalence–high risk’ to a ‘concentrated epidemic’ state, yet the forcefulness required for managing this silent escalation of HIV infected numbers is not being highlighted, as it should be. A more comprehensive review of the national strategy for HIV/AIDS would necessitate a system's thinking. For this purpose, the WHO's Health Systems Building Blocks have been discussed to analyse whether this framework can be employed to take some corrective measures. An extensive literature review in this regard helps to understand that the service delivery has to be responsive, but skilled human resources, a robust information system, an uninterrupted supplies and use of latest technology, adequate financing, and above all good governance at operational level are essential ingredients, which call for re-orienting the national programme today. Lack of coordination, capacity, and interventions with questionable sustainability pave a perilous path. Hitherto, the issue can be addressed by involving stakeholders from all levels of the society and managing the void between policy and implementation. Furthermore, interventions that focus on the long-term future are imperative to combat the menace threatening human lives.

From the emergence of the first case in 1987 to an alarming toll of more than 83,000 HIV/AIDS cases, Pakistan has moved to the category of a concentrated epidemic country (1, 2). In spite of its relatively conservative appearance, the society is harbouring large numbers of injecting drug users (3), nurturing an unregulated sex industry (4), allowing poorly screened blood products (5), permitting malpractice by unregistered medical practitioners, and reflecting a chronic incapacity of the health system to provide sustainable preventive programmes. Adding to the gravity of the situation are the complex social networks between the high-risk groups and the general population (6). In 2011, as a result of a constitutional amendment, the government of Pakistan devolved the powers of the federal administrative structure to its provincial counterparts. Although the aim was to transfer authority in certain concurrent portfolios to sub-national level, this step created a substantial operational void and grey areas regarding the role, capacity, and authority of the newly designated offices, especially in the smaller provinces (7). Perhaps, this decentralisation was done without a reality check on the level of preparedness of the provinces. National programme, federal ministry, provincial departments, NGOs, civil society, and the health service delivery outlets have been working in silos. Nevertheless, a collective and concerted nature of effort with regard to combating the epidemic is not visible. A more comprehensive review of the national strategy for HIV/AIDS would necessitate a system's thinking. For this purpose, the WHO's Health Systems Building Blocks have been discussed to analyse whether this framework can be employed to take some corrective measures (8). An extensive literature review in this regard helps to understand how the responsiveness of the health system can be improved.

## Methods

To build our main set of arguments, we have chosen the WHO's Health Systems Building Blocks to elaborate the present situation with regards to the response to the HIV epidemic in Pakistan. Each building block is discussed separately in this article based on the information available in the existing literature. To explain the situation according to each building block, a literature search was conducted over the internet, using nomenclature of the building blocks, that is, service delivery, health workforce, information system, drugs-supplies-technologies, financing, and leadership, and governance in HIV/AIDS sector. The online library of Pakistan's National AIDS Control Programme and websites of The Global Fund to Combat AIDS, Tuberculosis and Malaria and the World Health Organization were used for our literature search. We consulted Google Scholar, for the same Medical Subject Headings (MeSH) terms, and the full texts of articles of our choice were retrieved from Medline/PubMed, where available.

## Findings

Like any other developing country, in Pakistan, first level care is mostly sought in the private sector, because of distrust in the quality of care in the government system, limited working hours, shortage of trained human resources, and dearth of supplies (9). A meagre portion of gross domestic product (GDP) and a minimal developmental expenditure on health have been incurred persistently over the last two decades in the public sector health system of Pakistan (10). Most of the proportion in the allocation has chiefly gone to the recurring costs of a few big hospitals, and therefore the vertical programmes such as National HIV/AIDS Control Programme are primarily supported by the bilateral donors like Global Fund (11). By and large, the health system has been weak in all areas from service delivery, human resources, information system, to matters pertaining to governance and leadership. We used WHO's framework of building blocks for health system strengthening and analysed the health system of Pakistan for taking stock of the responsiveness towards the threat of HIV/AIDS in the country. Below is an account of each building block with its issues and proposed solutions.

### Service delivery

HIV treatment is available at designated anti retroviral therapy (ART) centres. Nevertheless, the treatment, care, and support facilities are available in the large metropolitans only (12). According to latest estimates, Pakistan has around 90,000 people living with HIV (PLWHIV) out of which 7,819 registered with the NACP. ART coverage is only 9.8% while ART coverage of injectable drug users (IDUs) with HIV is less than 1%. Moreover, the health services provided by the unregistered medical practitioners and the unsafe injection practices contribute significantly to the spread of HIV (13). A greater thrust on the prevention of HIV is the need, besides strengthening the curative services. Health education using popular mass media is a useful strategy to raise awareness among the general public, as well as the vulnerable segments of population. Regulation of medical practice has to be more stringent to curb the spread of HIV through the unhygienic and septic environment of many of the private and government clinics.

### Health workforce

The health workforce for HIV/AIDS is not only deficient in number but also lacks the required set of skills needed for dealing with HIV/AIDS cases (14).

The need for future planning regarding an improved health workforce is strong because the skill set needs to be upgraded with the changing patterns that HIV spreads in the country. There must be a continuing medical education programme for postgraduate trainees as well as the general practitioners to keep them abreast with the latest developments in the HIV/AIDS sector. More incentives for the workers would definitely keep their motivation alive for serving the difficult cases. Rural-based service rotations can be made a policy point so as to cater for the unequal distribution of doctors and nurses at the primary health care level.

### Information system

The public sector health management information system (HMIS) system lacks completeness, authenticity and therefore needs complete revamping. Moreover, the information from the vertical programmes like HIV/AIDS needs a meaningful integration in HMIS for timely decision making (15).

### Medical products, vaccines and technologies

The individuals coming in for HIV testing and for registered cases seeking treatment from public facilities are given free treatment. However, at times, a serious shortage of anti retroviral vaccine (ARV) is observed because of poor future planning. Procurement guidelines, schedules and supply chain must be revisited and adapted in the wake of the changing burden of disease and transition of roles between the national and provincial programme offices.

### Financing

In Pakistan, the HIV/AIDS programme has always depended on the donors and extra mural funding. The bilateral and multilateral donors and other development partners have invested to address the HIV threat in Pakistan. The Global Fund's total pledge to HIV/AIDS in Pakistan is US$ 37,523,500. However, the grant performance has been rated as inadequate and did not meet the expectations (16). In all times, capacity of the national programme to coordinate with provinces has been a challenge, and this has been even grimmer after the 2011 devolution. Accountability has to be instituted strictly at all levels and in all components of the programme. The private sector must be involved in the sharing of responsibility and risk pooling.

### Leadership and governance

Leadership of the national programme has been a complex affair. Political transition, administrative decentralisation, and donor dependency have their own implications. Governance reflects certain weaknesses such as shortage of supplies, irregularities in the use of funds, and violation of rules in appointments and procurements (17). Besides the national programme, almost 50 NGOs have been working on the cause with their own defined goals and objectives and modus operandi with least coordination with the country plan. This is where a health system's thinking and a strong political will emerges as the need of the hour.

## Discussion

The health system in Pakistan reflects a definite lag in terms of achieving the millennium development goals (MDGs) of maternal and child health as well as of tuberculosis, malaria, and HIV/AIDS. It would be critical to carry out a thorough stocktake to analyse the performance of the health system of Pakistan, in light of planned and achieved targets of HIV/AIDS. Such a gap analysis will give an account of gaps and flaws in the system, responsible for the current state of affairs. This is an opportune time to carry out this exercise, because the ongoing reforms will have the chance to look into deficiencies of all building blocks of the health system (18). The WHO's Health Systems Building Blocks framework encompasses all essential components that, directly and indirectly, affect the process of systems thinking, gap identification, and later strengthening of the action plan. There are various models based on health system's approach which have been oft-advocated, but some of these focused on the financial aspect (19), while some looked at the service delivery dynamics (20). We have endeavoured to critically analyse each and every aspect of the health system, and presented the solutions which might help in building a logical strategy, with all the building blocks of health systems strengthening, to cope with the threat of HIV/AIDS.

## Conclusion

All stakeholders working to win the battle against HIV/AIDS must be convinced to work and employ health system strengthening building blocks if desired results are to be achieved. The national programme of the government, NGOs, and the donors must invest their energies to review and strengthen all aspects from financing to governance, and from service delivery to human resources, with evidence-based decision making, using authentic and reliable information systems.
